# Enhanced Cathode Performance in Pr_0.5_Sr_0.5_FeO_3−δ_ of Perovskite Catalytic Materials via Doping with VB Subgroup Elements (V, Nb, and Ta)

**DOI:** 10.3390/ma17071635

**Published:** 2024-04-03

**Authors:** Hongfei Chen, Zhe Lü, Yujie Wu

**Affiliations:** School of Physics, Harbin Institute of Technology, Harbin 150001, China

**Keywords:** perovskite, stability, cathode catalyst, oxygen adsorption, impedance

## Abstract

Perovskite-style materials are cathode systems known for their stability in solid oxide fuel cells (SOFCs). Pr_0.5_Sr_0.5_FeO_3−δ_ (PSF) exhibits excellent electrode performance in perovskite cathode systems at high temperatures. Via VB subgroup metals (V, Nb, and Ta) modifying the B-site, the oxidation and spin states of iron elements can be adjusted, thereby ultimately adjusting the cathode’s physicochemical properties. Theoretical predictions indicate that PSF has poor stability, but the relative arrangement of the three elements on the B-site can significantly improve this material’s properties. The modification of Nb has a large effect on the stability of PSF cathode materials, reaching a level of −2.746 eV. The surface structure of PSF becomes slightly more stable with an increase in the percentage of oxygen vacancy structures, but the structural instability persists. Furthermore, the differential charge density distribution and adsorption state density of the three modified cathode materials validate our adsorption energy prediction results. The initial and final states of the VB subgroup metal-doped PSF indicate that PSFN is more likely to complete the cathode surface adsorption reaction. Interestingly, XRD and EDX characterization are performed on the synthesized pure and Nb-doped PSF material, which show the orthorhombic crystal system of the composite theoretical model structure and subsequent experimental components. Although PSF exhibits strong catalytic activity, it is highly prone to decomposition and instability at high temperatures. Furthermore, PSFN, with the introduction of Nb, shows greater stability and can maintain its activity for the ORR. EIS testing clearly indicates that Nb most significantly improves the cathode. The consistency between the theoretical predictions and experimental validations indicates that Nb-doped PSF is a stable and highly active cathode electrode material with excellent catalytic activity.

## 1. Introduction

Solid oxide fuel cells (SOFCs) perform coordinated work using various components [1,2,3,4,5,6,7,8,9]. The basic components of a battery include three parts. An electrolyte enables the transfer of ions between the cathode and anode and effectively separates the electrodes (cathode and anode) [10,11,12,13]. Among these sections, in the cathode, oxygen undergoes a reduction reaction on the electrode side to generate electrons, ultimately producing oxygen ions.

Tracing the development history of cathode systems, perovskite-type materials occupy the dominant position [14,15,16,17,18,19,20]. In the classic perovskite system (ABO_3_), A-site oxygen ions appear in a twelve-coordinate arrangement, while B-site ions have a six-coordinate arrangement. At different oxygen vacancy concentrations, Sr_n_Fe_n_O_3n−1_ materials exhibit four crystallographic phases of metal oxides. The final crystal structure is determined through X-ray diffraction (XRD). Specifically, when *n* = 2, it exhibits a Brownmillerite-type crystal structure; when *n* = 4, the material exhibits an orthorhombic structure; when *n* = 8, it exhibits a tetragonal structure; and when n approaches infinity, it exhibits a simple cubic structure [21]. Bassat et al. found that Pr has a strong oxygen diffusion ability, and A-site doping occurs in the system, further enhancing the oxygen diffusion [22]. Bhoga et al. investigated the introduction of the A-site Sr doping of perovskite materials and found that it changes the oxygen interface reaction. The polarization curve decreases to 0.3 Ω cm^2^ at 700 °C [23].

In cathode material systems, iron-based materials have received extensive research attention due to their good stability, suitable coefficient of thermal expansion, and lower cost. SrFeO_3−δ_-based materials are the most widely studied for cathodes in iron-based systems. Other researchers have shown that the structure of SrFeO_3−δ_ varies significantly with the temperature and oxygen partial pressure [24,25,26,27,28,29,30,31,32,33,34,35,36]. Jiang et al. introduced high-valence Nb into Fe and found that the modification of Nb enhanced the stability of the crystal and effectively avoided the phase transition. At the same time, it improved the catalytic activity [25]. Furthermore, several studies have shown that introducing Bi as an element at the A-site can also enhance the stability in the system [30]. Based on this, research on materials with simultaneous doping at both the A- and B-sites has been conducted [25,26]. To minimize the polarization loss of cathode materials during operation, it is essential for them to possess high electrical conductivity, thereby reducing the ohmic loss during electron transport.

As mentioned, the main issue with the perovskite system is the lack of structural stability due to the tendency of the crystal to undergo phase transition. Therefore, it is crucial to enhance the structural stability of materials and effectively prevent crystal phase transitions, while ensuring the cathodic catalytic activity of the structural system. In ABO_3_, the size of the BO_6_ octahedra depends on the matching of the ionic radii with the A-site ions; otherwise, the structural tilting of the BO_6_ structure will occur. Crystal structure distortion leads to a reduction in the symmetry of ABO_3_, transitioning from a high- to a low-symmetry crystal system. For example, it may change from cubic to orthorhombic or from orthorhombic to monoclinic. The transition of the crystal structure towards reduced symmetry can lead to defects in the ABO_3_ lattice, with many oxygen vacancies. This can affect the connectivity of BO_6_ units, leading to decreased stability. In terms of overcoming this issue, VB subgroup metal modification is the most effective means of adjusting the structural stability and cathode conductivity of Pr_0.5_Sr_0.5_FeO_3−δ_ (PSF). Although no experimental or theoretical reports indicate that the VB metal doping of PrSrFeO_3−δ_ can alter its structural stability, it has been proven that B-site regulation can effectively enhance the physicochemical properties of cathode materials. The most common B-site modifications can be classified into three categories. The first is to introduce high-valence state ions to stabilize the crystal structure and enhance the resistance to carbon deposition [37,38]. The second is to introduce transition elements to affect the conductivity of the corresponding cathode material [39]. Lastly, doping with noble metals is conducted to promote the surface exchange efficiency of oxygen, thereby improving the physicochemical properties of the cathode material [40,41]. A study by Yao et al. found that when Ta and Mo are singly or co-doped into SrFeO_3−δ_, the electrochemical catalytic performance is better than that caused by single doping. The synergistic effect produced by co-doping increases the number of oxygen vacancies in the material, ultimately improving the cathode electrode performance. The output power at 800 degrees Celsius can reach 931 mW·cm^−2^ [42]. Therefore, introducing suitable ions into the B-site is an appropriate and effective strategy for cathode material modification.

In this theoretical and experimental work, we primarily focus on doped Pr_0.5_Sr_0.5_FeO_3−δ_ cathode materials with VB subgroup metals. This includes theoretical calculations (surface formation energy, oxygen adsorption properties, charge distribution states, and d-band center movement trends) and experimental research (material synthesis and catalytic performance testing) using computational predictions. We find that Nb-doped Pr_0.5_Sr_0.5_FeO_3−δ_ exhibits promising potential as a cathode catalyst, attributed to its adsorption properties, significant charge transfer distribution condition, and d-band shifts. Meanwhile, by utilizing optimization methods to regulate high-valence state ions at the B-site, we develop new cathode materials with high stability and excellent electrode physicochemical performance: Pr_0.5_Sr_0.5_FeO_3−δ_-based materials. The adsorption energy value of Nb-doped PSF is adjusted to −2.746 eV. In addition, the polarization resistance of Nb-doped PSF decreases at different temperatures. Our work highlights that the Fe-site can effectively determine the cathode catalytic property of PrSrFeO_3−δ_.

## 2. Experimental and Calculation Methods

### 2.1. Experimental Methods

The more complex component, PSFN, is used as an example. The Pr_0.5_Sr_0.5_Fe_0.9_Nb_0.1_O_3−δ_ powder cathode systems were created using the modified sol–gel method. Pr_6_O_11_, Fe(NO_3_)_3_·9H_2_O, and SrCO_3_ were placed in a nitric acid solution, and a standard amount of EDTA was placed in high-quality deionized water (using the same process as for citric acid). The molar ratio of EDTA, metal cations, and citric acid was set as 1:1:1. Finally, an appropriate amount of ammonia solution was poured into the solution, and the pH of the mixture was adjusted to 7–8. After 2 h stirring, a black precursor was formed. Then, the burnt remains were cooled and kept for later use; after this, the solution was continuously calcined for 3 h to obtain the cathode powder at 1100 °C. The same process was performed to obtain the PSF cathode powder.

XRD tests were performed using a Rigaku Smartlab X-ray diffractometer (Akishima, Japan) with Cu target Kα radiation (λ = 0.15418 nm) at an angle range of 20°–80°. In addition, the crystal microstructures of the powder sample were studied using an transmission electron microscope (Tecnai-G2-F30, FEI Corporation, Valley City, ND, USA). Meanwhile, the microstructure was visualized using a scanning electron microscope (JSM-7001F, JEOL, Akishima, Japan).

The PSFN powder was mixed with ethyl cellulose pine oil alcohol binder (with a mass ratio of pine oil alcohol to ethyl cellulose of 9:1) in a 1:1 mass ratio to prepare a PSFN electrode paste, and then the PSFN electrode paste was evenly applied on both surfaces of the dense La_0.8_Sr_0.2_Ga_0.8_Mg_0.2_O_3−δ_ (LSGM) electrolyte circular disc green body. Pr_0.5_Sr_0.5_Fe_0.9_Nb_0.1_O_3−δ_ was added to both sides of the surface of the LSGM electrolyte via the brush coating method, heated to 1100 °C for three hours, and processed at 800 °C Celsius for 1 h to form a porous silver current collector layer to complete the symmetric cell (PSFN | LSGM | PSFN), with the LSGM electrolyte as the support and the PSFN material as the electrode. The effective area was approximately 0.28 cm^2^. The cathode slurry was slightly thinner than 20 µm.

To examine the pre-made symmetric cells, electrochemical impedance spectroscopy (EIS) was adopted using a Bistat potentiostat (VSP, Biologic, France). The electronic conductivity of the electrode material was measured as the temperature variation in air using a four-point probe electrode.

### 2.2. Calculation Methods

Density functional theory (DFT) was realized with the VASP code [43,44]. The calculation method was represented by GGA + U (*U*_Fe,d_ = 5.3 eV, *U*_V,d_ = 5.3 eV, *U*_Nb,d_ = 5.3 eV, and *U*_Ta,d_ = 5.3 eV) [45,46,47]. During stimulation, the energy convergence and the cutoff energy were 1.0 × 10^−6^ eV and 520 eV, respectively. In addition, cathode surface vacuum layers were added along the z-direction (20 Å). Each PSF (1∙1∙1 unit cell) cathode contained 20 atoms. The Brillouin zone of the VB subgroup metal-doped Pr_0.5_Sr_0.5_FeO_3−δ_ k-point mesh surface was 6 × 6 × 1. All the force criteria were set to 0.05 eV/Å. Moreover, the maximum stresses were all measured at 0.1 GPa.

## 3. Results and Discussion

Following the calculations and experimental methods described in the previous section, we first conducted VASP calculations to separately predict the stability of the pure V and the Nb and Ta Pr_0.5_Sr_0.5_FeO_3−δ_ cathodes to identify the cathodes with good stability. Next, we performed predictive calculations on the V-, Nb-, and Ta-doped Pr_0.5_Sr_0.5_FeO_3−δ_ cathodes regarding adsorption, cathode charge transfer, the cathode surface d-band theory, and the initial and final states of the cathode, aiming to identify the Ta-doped Pr_0.5_Sr_0.5_FeO_3−δ_ cathode with relatively prominent catalytic activity. Finally, we performed experimental verification.

### 3.1. Surface Material Stability of VB Metal-Doped PSF

#### 3.1.1. Surface Formation Energy of VB Metal-Doped PSF

**Figure 1:**
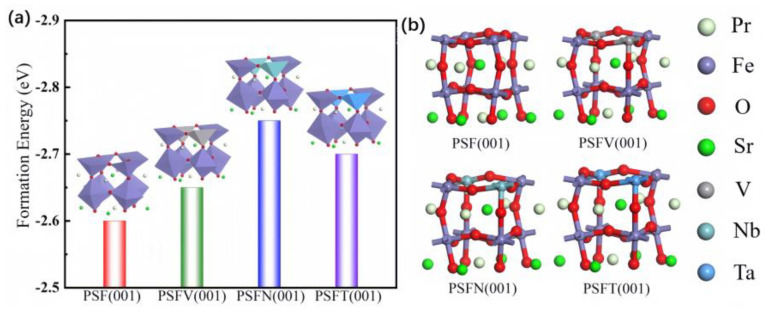
PSF (001), PSFV (001), PSFN (001), and PSFT (001) materials: (**a**) the surface formation energy and (**b**) the optimized model.

The surface forming energy of the Pr_0.5_Sr_0.5_FeO_3−δ_ surface systems is shown in Figure 1. Doping with the VB subgroup metals was achieved by substituting one Fe atom with V, Nb, and Ta in a complete PSF (001) unit cell. The concentration of each dopant element in the model was 25% (1/4). The pristine Pr_0.5_Sr_0.5_FeO_3−δ_ had an unstable surface formation energy (−2.60 eV), which was consistent with its previous stability problems. The stability was regulated by the B-site doped Pr_0.5_Sr_0.5_FeO_3−δ_. The VB subgroup metal-doped PSF had a different atomic arrangement, with the stability of an open surface structure, being superior to that of a densely packed atomic structure. The surface formation energy was obviously improved by the VB subgroup metal doping. Among them, Nb most effectively improved the Pr_0.5_Sr_0.5_FeO_3−δ_ surface’s stability, which was increased to −2.75 eV. The increase in surface forming energy improved the Pr_0.5_Sr_0.5_FeO_3−δ_ cathode’s stability. After the introduction of the VB subgroup metals, PSF surface enhancement occurred due to the expansion of the crystal after the introduction of these three elements, leading to a change in the ionic radius and the redistribution of the surface charge of the PSF layer, ultimately achieving the lowest energy (most stability) state out of the VB subgroup metal-doped PSF cathode systems. Therefore, doping with V, Nb, and Ta resolved the poor stability of PSF, so we only needed to study further the cathode catalysis of the V-, Nb-, and Ta-doped PSF.

#### 3.1.2. Oxygen Vacancy (VO) Stability on the Pr_0.5_Sr_0.5_FeO_3−δ_ Cathode Surface

**Figure 2:**
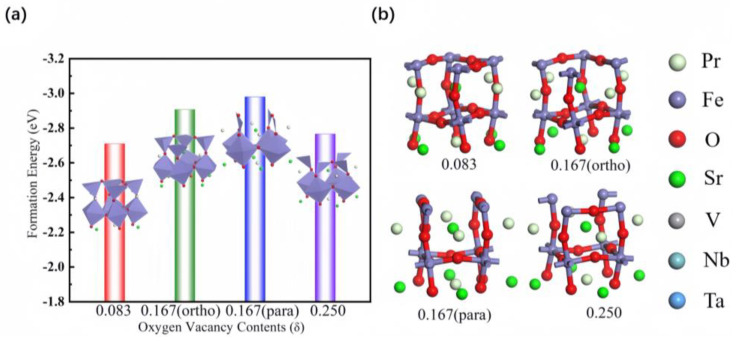
The PSFO_2.917_, ortho-PSFO_2.833_, para-PSFO_2.833_, and PSFO_2.750_ surface materials. (**a**) The VO formation energy and (**b**) optimized lattice.

Based on a previously optimized surface model of PSF, further optimization was carried out for VO defects of different concentrations on the PSF surface, in order to calculate the formation energy of the Pr_0.5_Sr_0.5_FeO_3−δ_ VO system. By removing one O atom, two O atoms (ortho-position and oppo-position), or three O atoms in a complete PSF (001) unit cell, we could introduce concentrations of 8.3% (1/12), 16.7% (2/12), and 25% (3/12) VO, respectively. The formation energies of Pr_0.5_Sr_0.5_FeO_3−δ_ with different VO concentrations (0.083, ortho −0.167, para −0.167, and 0.250) on the (001) surface are shown in Figure 2. The VO systems (various concentrations) of pure Pr_0.5_Sr_0.5_FeO_3−δ_ showed that the para −0.167 VO percentage had the lowest formation energy (−2.980 eV) of Pr_0.5_Sr_0.5_FeO_3−δ_, with the most stable optimized result. Pr_0.5_Sr_0.5_FeO_3−δ_ with VO maintained a perovskite framework structure, which was embodied in the lattice, and the entire lattice system did not deform. The presence of VOs in the lattice could trigger Pr_0.5_Sr_0.5_FeO_3−δ_ surface lattice reconstruction, thereby enhancing the stability of the Pr_0.5_Sr_0.5_FeO_3−δ_ (001) surface.

### 3.2. PSF (001) Cathode Surface

#### 3.2.1. Catalytic Properties

**Figure 3:**
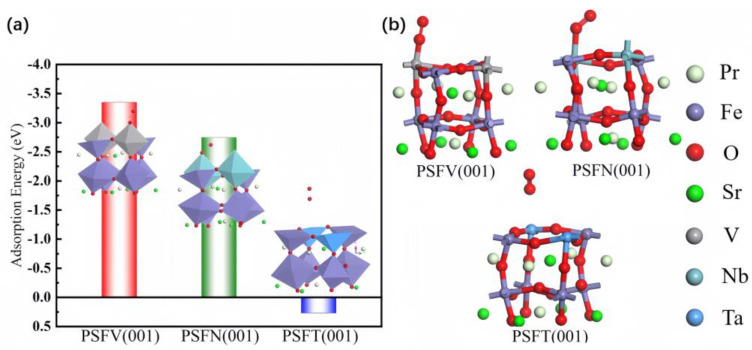
PSFV (001), PSFN (001), and PSFT (001) surface materials: (**a**) adsorption energy and (**b**) optimum adsorption model.

Adsorbing oxygen on the previously optimized crystal surface model of the VB subgroup metal-doped PSF allowed the stable adsorption of the cathode fuel (O_2_). The adsorption energy of the VB subgroup metal-doped PSF cathode surface is shown in Figure 3. The catalysis principle of the cathode is defined as follows:

(1) $$\frac{1}{2}O_{2}+2e^{-}\to 2O^{2-}$$

This catalytic reaction mainly occurs on Pr_0.5_Sr_0.5_FeO_3−δ_ with a VB subgroup metal-doped cathode surface. The adsorption energies of different doping sites on the cathode were calculated for each type of Pr_0.5_Sr_0.5_FeO_3−δ_ with doped VB subgroup metals. To determine the optimal sites on the Pr_0.5_Sr_0.5_FeO_3−δ_ cathode surface systems, the adsorption energies of O_2_ on the three optimal adsorption sites of Pr_0.5_Sr_0.5_FeO_3−δ_ with doped VB subgroup metals were compared. The changes in the adsorption sites in the VB subgroup metal-doped PSF were mainly due to the incremental ionic radii of V, Nb, and Ta, resulting in the rearrangement of the surface atomic structure of the cathode. When oxygen molecules adsorb on the surface of a VB subgroup metal-doped PSF cathode, they shift from Fe to above V, Nb, and Ta.

The optimal cathode adsorption sites for VB subgroup metal doping are different. The best cathode adsorption sites are directly above the VB subgroup metal atom for oxygen. In terms of energy, the frontier orbitals of O_2_ interact with the valence band of the VB subgroup metal-doped PSF, reducing the potential energy of the O_2_ adsorption surface. In terms of bonding, O_2_ forms chemical bonds with the surface of the VB subgroup metal-doped PSF. In terms of geometry, the VB subgroup metal-doped PSF reconstructs the two-dimensional unit cell containing O_2_. The oxygen adsorption energies on the Pr_0.5_Sr_0.5_FeO_3−δ_ cathode surface doped with V, Nb, and Ta are −3.348 eV, −2.746 eV, and 0.268 eV, respectively. Positive adsorption energy on the Ta-doped cathode surface indicates that the Pr_0.5_Sr_0.5_FeO_3−δ_ cathode surface does not adsorb oxygen well, indicating poor catalytic performance. This result can be interpreted by the large ionic radius of Ta. If the oxygen adsorption energy on the Pr_0.5_Sr_0.5_FeO_3−δ_ cathode surface doped with V exceeds −3 eV, the Pr_0.5_Sr_0.5_FeO_3−δ_ cathode surface adsorbs oxygen too strongly, which can lead to the catalytic poisoning of the cathode surface. Compared to this, the O_2_ adsorption energy on the cathode doped with Nb is the most suitable, indicating that the PSFN surface is an ideal cathode catalyst.

**Figure 4:**
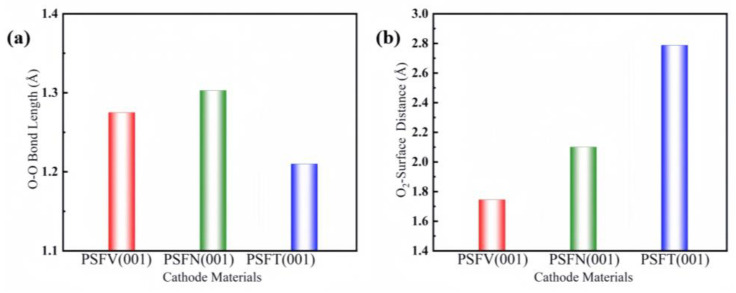
PSFV (001), PSFN (001), and PSFT (001) surface materials: (**a**) O–O bond distance and (**b**) O_2_–surface distance.

The O–O bond lengths (after breakage) are shown in Figure 4. After breakage, the O–O bond lengths of the VB subgroup metal-doped surface (001) are 1.275 Å, 1.303 Å, and 1.210 Å, respectively. The distances between the oxygen and the cathode surfaces of the VB subgroup metal-doped surface (001) are 1.745 Å, 2.101 Å, and 2.788 Å, respectively. The calculated oxygen adsorption energy and O–O bond lengths are consistent with the O_2_–surface distances.

#### 3.2.2. Charge Transfer Condition

The VB subgroup metal-doped surface (001) electronic transfer status was studied, and the cathode’s differential charge densities were calculated, which can be defined as follows:

(2) $$\Delta \rho =\rho_{sur-O_{2}}-\rho_{sur}-\rho_{O_{2}}$$

where $\rho_{\mathrm{sur}-O2}$ and $\rho_{sur}$ represent the O_2_ adsorption charge density of the cathode and the unadsorbed original surface, respectively. $\rho_{O2}$ denotes the charge density of the O_2_ molecule.

**Figure 5:**
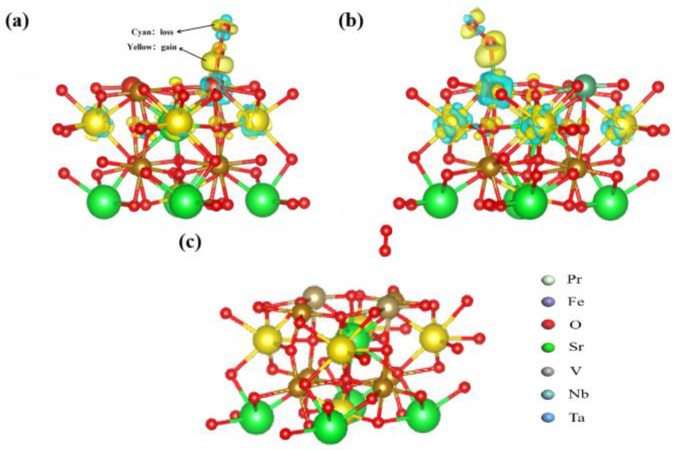
The differential charge density: (**a**) PSFV (001), (**b**) PSFN (001), and (**c**) PSFT (001) surface materials.

Figure 5 shows that the oxygen transported to the Ta-doped cathode surface (001) does not undergo electron transfer. The cyan and yellow parts indicate the losses and gains of electron clouds, respectively. In stark contrast, the oxygen on the V- and Nb-doped cathode surfaces (001) does not undergo electron transfer. The bonding portion of electrons from V to the cathode PSF surface is more concentrated, with higher stability in adsorption, and is also more concentrated near the V atoms directly beneath the oxygen. On the other hand, the electron concentration of Nb to the cathode surface PSF is lower, indicating that the adsorption is less stable compared to V doping. At the same time, the surface Fe and Nb atomic charges of the entire cathode PSF increase and decrease, indicating the redistribution of the charges throughout the entire PSF surface layer, rather than locally. This is in line with the previous oxygen catalytic performance.

#### 3.2.3. Adsorption Structure d-Band Center

**Figure 6:**
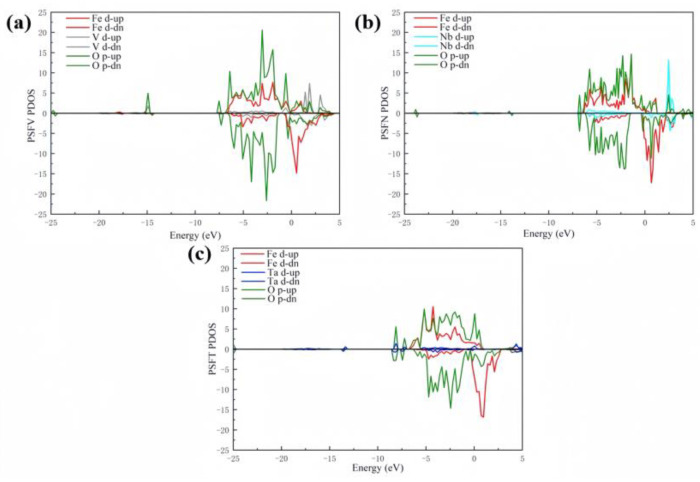
The PDOS: (**a**) PSFV (001), (**b**) PSFN (001), and (**c**) PSFT (001) surface materials.

The d-band of the VB subgroup metal-doped surface (001) triggers bonding–antibonding states on the perovskite cathode surface. The d-PDOS of the VB subgroup metal-doped cathode PSF surface is displayed in Figure 6. The adsorption state densities of V, Nb, and Ta are mainly determined by the d-states (3d, 3d, 4d, and 5d) of Fe, V, Nb, and Ta, as well as the 2p-state of O_2_, in the adsorbed molecules. The antibonding orbitals of O_2_ hybridize with the d-bands of Fe, V, Nb, and Ta, forming a conduction band that shifts to below the Fermi level and overlaps to create new bonding orbitals. Among them, the band above the Fermi level in the V-doped PSF cathode is the highest, indicating that minimal electron filling occurs in the antibonding band of the PSFT. In contrast, the opposite occurs for the Ta-doped PSF cathode. At the same time, the Nb-doped PSF cathode has a moderate result, with the band above the Fermi level being relatively large, indicating that less electron filling occurs in the antibonding band of the PSFT. Relative to Ta doping, the V and Nb metal-doped PSF (001) cathode surfaces have d-band center shifts. Therefore, V and Nb can effectively improve PSF (001) cathode surface catalysis.

#### 3.2.4. Initial and Final States of the Pr_0.5_Sr_0.5_FeO_3−δ_ (001) Doping System

**Figure 7:**
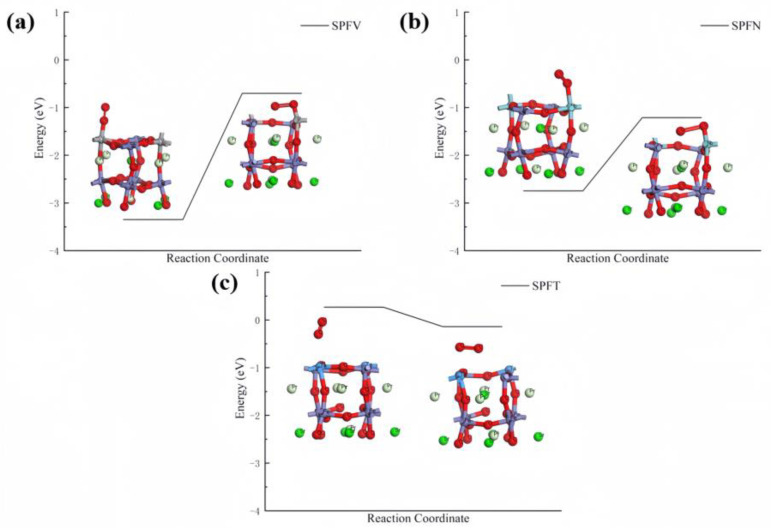
Catalysis reaction initial and final states: (**a**) PSFV (001), (**b**) PSFN (001), and (**c**) PSFT (001) surface materials.

Figure 7 shows the initial and final surface adsorption of the V-, Nb-, and Ta-doped Pr_0.5_Sr_0.5_FeO_3−δ_ cathodes. A decrease in the adsorption potential energy is achieved at the expense of electrons from the surface of the VB subgroup metal-doped PSF and O_2_ transfer from their original orbitals to new orbitals in the adsorption structure. The initial value of O_2_ adsorption on the surfaces of the four layers of atoms of V-doped Pr_0.5_Sr_0.5_FeO_3−δ_ is −3.348 eV. Subsequently, in the most exposed surface layer of V-doped Pr_0.5_Sr_0.5_FeO_3−δ_, the adjacent O (under the SOFC mechanism) in the cathode port is supplemented towards the anode port, causing the exposed surface of the cathode to undergo O loss between V and Fe. At this point, the adsorbed O–O gradually elongates after being adsorbed in gaseous form and is eventually adsorbed in the form of two oxygen atoms (O_1_ and O_2_) on the surface. One is located directly above the V atom, and the other is located directly above the oxygen vacancy, where O was originally missing. The final value of O_1_–O_2_ adsorption on the four layers of atoms in the V-doped Pr_0.5_Sr_0.5_FeO_3−δ_ cathode is −0.703 eV.

The initial value of O_2_ adsorption on the surfaces of the four layers of atoms of the Nb-doped Pr_0.5_Sr_0.5_FeO_3−δ_ is −2.746 eV. Subsequently, in the most exposed surface layer of the Nb-doped Pr_0.5_Sr_0.5_FeO_3−δ_ cathode, the O adjacent to Nb is supplemented towards the anode end, causing the exposed surface to undergo O loss between Nb and Fe. At this point, after the elongation of O–O, the most stable state is found to have one O atom located directly above the Nb atom and another above the oxygen vacancy, where O was originally missing. The most stable O_1_–O_2_ adsorption value on the four layers of atoms in the Nb-doped Pr_0.5_Sr_0.5_FeO_3−δ_ cathode is −1.213 eV. In a more unique case, Ta-doped Pr_0.5_Sr_0.5_FeO_3−δ_ shows almost no O_2_ adsorption in the initial state. After supplementing with an O atom towards the anode, the slight adsorption of −0.141 eV occurs between O_1_–O_2_ and the exposed surface.

Comparing the VB subgroup metal-doped PSF samples, V-doped Pr_0.5_Sr_0.5_FeO_3−δ_ is very easy to adsorb, but its final state shows less adsorption. In other words, the predicted cathode mechanism of V-doped Pr_0.5_Sr_0.5_FeO_3−δ_ is not very conducive to the next steps of the reaction. The initial adsorption state of Nb-doped Pr_0.5_Sr_0.5_FeO_3−δ_ is relatively moderate, and the final state of O_1_–O_2_ adsorption is also moderately elongated and fractured. The predicted cathode mechanism of Nb-doped Pr_0.5_Sr_0.5_FeO_3−δ_ is the most conducive to further reactions. In contrast, Ta-doped Pr_0.5_Sr_0.5_FeO_3−δ_ is very different from V-doped Pr_0.5_Sr_0.5_FeO_3−δ_, where almost no adsorption occurs in the initial state, but the final state shows more adsorption. Therefore, the predicted Ta-doped Pr_0.5_Sr_0.5_FeO_3−δ_ cathode is also not conducive to the reaction, which is similar to V doping.

### 3.3. ORR Mechanism of Nb-Doped Pr_0.5_Sr_0.5_FeO_3−δ_ (001)

#### 3.3.1. ORR Mechanism of PSF

**Figure 8:**
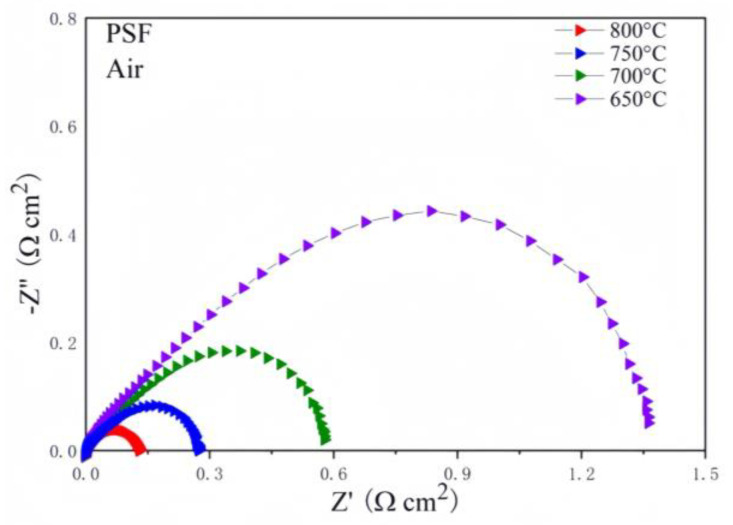
The cell performance in EIS of the PSF symmetric cell in air.

The experimental catalytic index of the oxygen reduction reaction (ORR) of the Pr_0.5_Sr_0.5_FeO_3−δ_ cathode system can be characterized using the electrochemical impedance spectra (EIS) of the PSF cathode in air. Utilizing the pre-encapsulated symmetric batteries PSF|LSGM|PSF, the electrochemical impedance of the Pr_0.5_Sr_0.5_FeO_3−δ_ cathode at four constant temperatures was measured in air. The EIS data are shown in Figure 8. The PSF cathode was tested with an R_p_ ranging from 800 to 650 degrees. The polarization impedance of Pr_0.5_Sr_0.5_FeO_3−δ_ greatly increased as the temperature decreased from 800 °C at 0.120 Ω cm^2^ to 650 °C at 1.310 Ω cm^2^. The electrical conductivity of the Pr_0.5_Sr_0.5_FeO_3−δ_ electrode continued to increase as the temperature rose. When the test temperature ranged from 800 °C to 650 °C, the polarization impedance R_p_ values of the PSF cathode were 0.12 Ω cm^2^ (800 °C), 0.27 Ω cm^2^ (750 °C), 0.59 Ω cm^2^ (700 °C), and 1.31 Ω cm^2^ (650 °C), respectively.

#### 3.3.2. Crystal Structure and Electronic Properties

**Figure 9:**
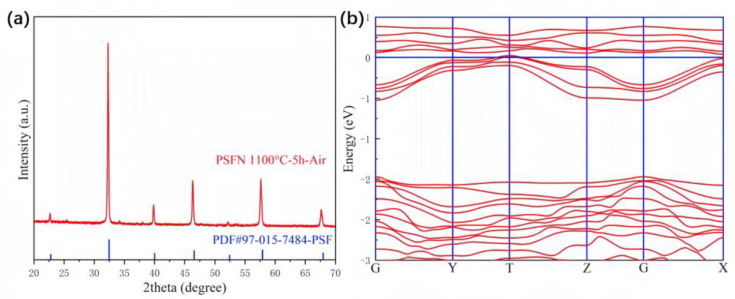
The PSFN bulk materials: (**a**) XRD and (**b**) electronic structure.

The XRD data for Pr_0.5_Sr_0.5_FeO_3−δ_ Nb doping are displayed in Figure 9a. The diffraction peaks of PSFN closely match those of PSF, and there are no impurities in PSFN, indicating that the Nb has been incorporated into the lattice of the PSF. The test results show that Nb-doped Pr_0.5_Sr_0.5_FeO_3−δ_ is an orthogonal system, which is completely consistent with the Nb-doped Pr_0.5_Sr_0.5_FeO_3−δ_ optimized model structure that we obtained. The XRD spectrum of PSFN reveals slight shifts in the main diffraction peaks, which can be compared to the corresponding peaks identified in the PDF card library for PSF, indicating the minimal structural expansion of the unit cell after Nb doping at the B-site. Table 1 lists the unit cell structural parameters of PSF and PSFN, showing that the newly formed system’s unit cell parameters also undergo minimal structural improvement after Nb doping. In a reducing atmosphere, due to O- being lost in the lattice, the oxidation state of iron shifts from high (Fe^3+^) to low (Fe^2+^), with a trivalent iron ion radius (Fe^3+^: 0.645 Å) that is slightly smaller than that of divalent iron ions (Fe^2+^: 0.78 Å), resulting in the unit cell parameters of PSFN being slightly larger than those measured before the reduction.

The Nb-doped Pr_0.5_Sr_0.5_FeO_3−δ_ band structure predicted theoretically with GGA + U is presented in Figure 9b. The band structure of Nb-doped Pr_0.5_Sr_0.5_FeO_3−δ_ displays a semi-metallic state. This means that there is a partial overlap between the energy bands (conduction and valence) of Nb-doped Pr_0.5_Sr_0.5_FeO_3−δ_.

#### 3.3.3. ORR Mechanism

**Figure 10:**
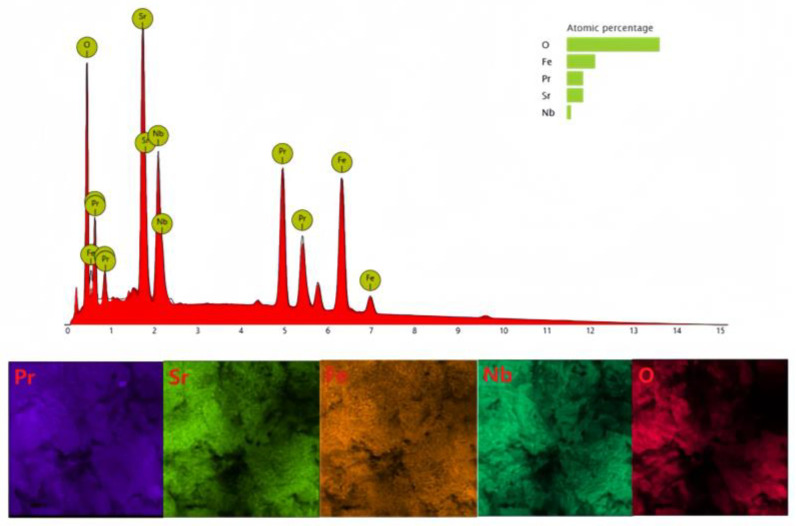
The cell performance in EDX of the PSFN symmetric cell in air.

The EDX result of Nb-doped Pr_0.5_Sr_0.5_FeO_3−δ_ is displayed in Figure 10. The Fe/Nb elemental ratio is approximately 9.06:1, indicating the stoichiometric ratio for the PSFN cathode composite manufactured for experimental testing in air. The occurrence of a low-angle deviation in the Nb-doped Pr_0.5_Sr_0.5_FeO_3−δ_ main diffraction peak indicates that the crystal structure expands after Nb doping.

**Figure 11:**
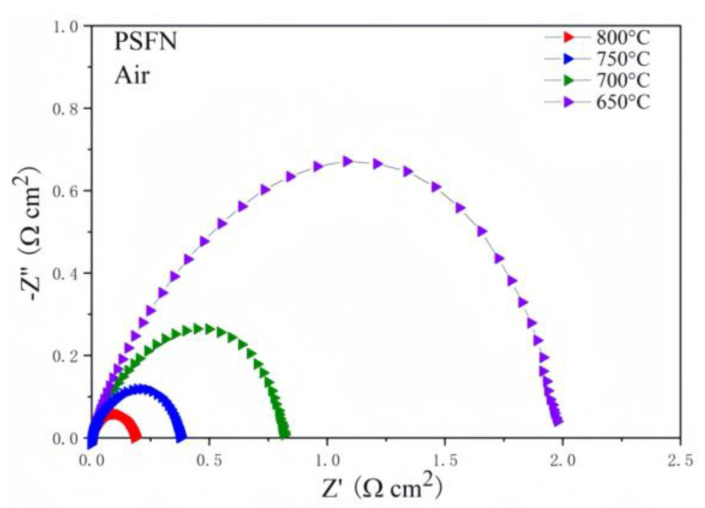
The cell performance in EIS of the PSFN symmetric cell in air.

The pre-encapsulated symmetric batteries PSFN | LSGM | PSFN were used again, and the Nb-doped Pr_0.5_Sr_0.5_FeO_3−δ_ cathode’s EIS at four constant temperatures was measured under ambient conditions in air. Figure 11 displays the symmetrical impedance of Nb-doped Pr_0.5_Sr_0.5_FeO_3−δ_. To enhance the activation ability of the PSFN cathode, the polarization of the current on the (001) surface of the Nb-doped Pr_0.5_Sr_0.5_FeO_3−δ_ cathode becomes dominant, and heating accelerates the polarization process via the (001) surface on the PSFN cathode.

The PSFN cathode was tested using an R_p_ ranging from 800 degrees to 650 degrees. The polarization impedance of Nb-doped Pr_0.5_Sr_0.5_FeO_3−δ_ greatly increased as the temperature decreased from 800 °C at 0.184 Ω cm^2^ to 650 °C at 2.078 Ω cm^2^. The electrical conductivity of the Nb-doped Pr_0.5_Sr_0.5_FeO_3−δ_ electrode continued to increase as the temperature rose. When the test temperature ranged from 800 °C to 650 °C, the polarization impedance R_p_ values of the PSFN cathode were 0.19 Ω cm^2^ (800 °C), 0.37 Ω cm^2^ (750 °C), 0.79 Ω cm^2^ (700 °C), and 2.07 Ω cm^2^ (650 °C), respectively. The PSFN cathode polarization impedance value Rp increased as the temperature decreased, indicating that the PSFN cathode material also exhibited good ORR experimental catalytic activity.

Furthermore, of the two cathode catalytic materials, PSF and PSFN, PSF exhibits stronger catalytic activity, but it is highly susceptible to decomposition at high temperatures, making it unstable. On the other hand, the introduction of Nb to PSFN enhances its stability, allowing the ORR activity to be relatively well maintained.

**Figure 12:**
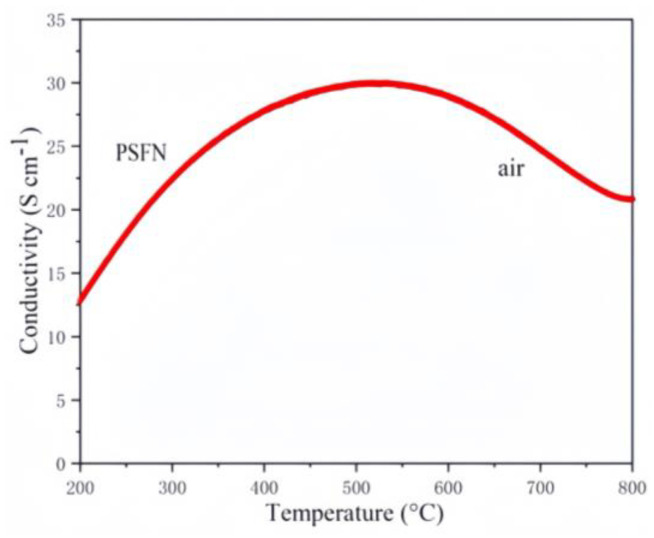
The conductivity in air: PSFN cathode materials.

The electrical conductivity of PSFN is shown in Figure 12. In the initial temperature range, the conductivity of PSFN in air increases nonlinearly with the temperature until it reaches a maximum of 29.6 S cm^−1^ at 520 °C, at which point the PSFN material undergoes a semi-metal transition. In the subsequent temperature range, the conductivity decreases nonlinearly until it reaches 22.5 S cm^−1^ at 800 °C. The charge transport in PSFN mainly occurs through hopping conduction along the Fe–O–Nb bonds. At high temperatures, the valence state at the B-site decreases, accompanied by an increase in oxygen vacancies (VO), hindering the transport of charge carriers and leading to a decrease in conductivity. The conductivity of PSFN is consistent with the previous EIS test results, both indicating that PSFN is a cathode material with good catalytic performance.

## 4. Conclusions

To summarize, using calculations and experiments, we integrally researched the VB subgroup metal V-, Nb-, and Ta-doped Pr_0.5_Sr_0.5_FeO_3−δ_ cathode. The simulation shows that Nb-doped Pr_0.5_Sr_0.5_FeO_3−δ_ has a value (−2.746 eV) that does not poison the cathode and exhibits enough cathode activity for a cathode reaction, and oxygen undergoes charge transfer with Nb-doped PSF, which promotes the charge flow distribution in the adsorption structure. Simultaneously, the d-band center shift of Nb-doped Pr_0.5_Sr_0.5_FeO_3−δ_ leads to an enhancement in the cathode during the SOFC reaction. In the V-, Nb-, and Ta-doped PSF samples, Nb-doped Pr_0.5_Sr_0.5_FeO_3−δ_ exhibits the easiest transition from the initial to the final state for the surface O_2_ adsorption reaction. In addition, the results of Nb-doped Pr_0.5_Sr_0.5_FeO_3−δ_ XRD and structural optimization confirm that it has an orthorhombic perovskite structure. Furthermore, the impedance of the Nb-doped Pr_0.5_Sr_0.5_FeO_3−δ_ symmetrical cell indicates that an Rp of 0.184 Ω cm^2^ can be achieved with a running display temperature of 800 °C. Of the two cathode catalytic materials, PSF and PSFN, PSF exhibits stronger catalytic activity, but it is highly susceptible to decomposition at high temperatures, making it unstable. Nevertheless, the introduction of Nb to PSFN enhances its stability, allowing the ORR activity to be relatively well maintained. This theoretical and experimental work shows that Nb can effectively improve the Pr_0.5_Sr_0.5_FeO_3−δ_ cathode’s catalytic performance.

## Figures and Tables

**Table 1:** Lattice parameters and unit volume of PSF and PSFN.

Materials	a (Å)	b (Å)	c (Å)	Unit Volume (V, Å^3^)
PSF	5.487	7.761	5.489	233.798
PSFN	5.542	7.820	5.546	240.494

## Data Availability

Data are contained within the article.

## References

[1] Ismael I., El-Fergany A.A., Gouda E.A., Kotb M.F. (2023). Cooperation Search Algorithm for Optimal Parameters Identification of SOFCs Feeding Electric Vehicle at Steady and Dynamic Modes. Int. J. Hydrogen Energy.

[2] Yokokawa H., Sakai N., Horita T., Yamaji K., Brito M.E. (2005). Electrolytes for Solid- Oxide Fuel Cells. MRS Bull..

[3] Badwal S.P.S., Foger K. (1996). Solid Oxide Electrolyte Fuel Cell Review. Ceram. Int..

[4] Irshad M., Baber K., Butt M.S., Hanif M.B., Asad M., Ghaffar A., Rafique M., Hussain I., Al-Anazy M.M., Makarov H. (2023). Synergistic Role of Biomolecules and Bio-Chelating Agents in the Sustainable Development of an Efficient BaCe_0.97_M_0.03_O_3−δ_ (M = Sm, Gd) Perovskite Electrolyte for IT-SOFC. Ceram. Int..

[5] Basbus J.F., Cademartori D., Asensio A.M., Clematis D., Savio L., Pani M., Gallus E., Carpanese M.P., Barbucci A., Presto S. (2024). Study of a Novel Microstructured Air Electrode/Electrolyte Interface for Solid Oxide Cells. Appl. Surf. Sci..

[6] Woo S.H., Song K.E., Baek S.-W., Kang H., Choi W., Shin T.H., Park J.-Y., Kim J.H. (2021). Pr- and Sm-Substituted Layered Perovskite Oxide Systems for IT-SOFC Cathodes. Energies.

[7] Bin Mohd Abd Fatah A.F., Murat M.N., Hamid N.A. (2021). LSCF-CuO as Promising Cathode for IT SOFC. J. Eng. Technol. Sci..

[8] Zamudio-García J., Porras-Vázquez J.M., Losilla E.R., Marrero-López D. (2024). Enhancing the Electrochemical Performance in Symmetrical Solid Oxide Cells through Nanoengineered Redox-Stable Electrodes with Exsolved Nanoparticles. ACS Appl. Mater. Interfaces.

[9] Sadykov V.A., Eremeev N.F., Sadovskaya E.M., Shlyakhtina A.V., Pikalova E.Y., Osinkin D.A., Yaremchenko A.A. (2022). Design of Materials for Solid Oxide Fuel Cells, Permselective Membranes, and Catalysts for Biofuel Transformation into Syngas and Hydrogen Based on Fundamental Studies of Their Real Structure, Transport Properties, and Surface Reactivity. Curr. Opin. Green Sustain. Chem..

[10] Dholabhai P.P., Adams J.B., Crozier P., Sharma R. (2010). A Density Functional Study of Defect Migration in Gadolinium Doped Ceria. Phys. Chem. Chem. Phys..

[11] Figueiredo F.M.L., Marques F.M.B. (2013). Electrolytes for Solid Oxide Fuel Cells. WIREs Energy Environ..

[12] Kilner J.A., Burriel M. (2014). Materials for Intermediate-Temperature Solid-Oxide Fuel Cells. Annu. Rev. Mater. Res..

[13] Jacobson A.J. (2010). Materials for Solid Oxide Fuel Cells. Chem. Mater..

[14] Pang S., Yang G., Su Y., Xu J., Shen X., Zhu M., Wu X., Li S., Chen C. (2019). A-Site Cation Deficiency Tuned Oxygen Transport Dynamics of Perovskite Pr_0.5_Ba_0.25−x_Ca_0.25_CoO_3−δ_ Oxide for Intermediate Temperature Solid Oxide Fuel Cells. Ceram. Int..

[15] Adler S.B. (2004). Factors Governing Oxygen Reduction in Solid Oxide Fuel Cell Cathodes. Chem. Rev..

[16] Teketel B.S., Beshiwork B.A., Desta H.G., Wang S., Li Z., Luo X., Workneh G.A., Lin B. (2024). Ta/Nb Doping Motivating Cubic Phase Stability and CO_2_ Tolerance of Sr_2_Co_1.6_Fe_0.4_O_6−δ_ Double Perovskite for High-Performing SOFC Cathode. Ceram. Int..

[17] Woo S.H., Yang H.J., Kim Y. (2024). Investigation of the Effect of Off-Stoichiometric Composition on Oxygen Transport in Layered Perovskite Materials for SOFC Cathode. Mater. Lett..

[18] Mishchenko D.D., Arapova M.V., Bespalko Y.N., Vinokurov Z.S., Shmakov A.N. (2023). In Situ XRD and TGA/DTA Study of Multiphase La- and Nd-Substituted Pr_2_NiO_4_ under IT-SOFC Cathode Operating Conditions. J. Alloys Compd..

[19] Priya S.D., Nesaraj A.S., Selvakumar A.I. (2021). Facile Wet-Chemical Synthesis and Evaluation of Physico-Chemical Characteristics of Novel Nanocrystalline NdCoO_3_-Based Perovskite Oxide as Cathode for LT-SOFC Applications. Bull. Mater. Sci..

[20] Siebenhofer M., Nenning A., Wilson G.E., Kilner J.A., Rameshan C., Kubicek M., Fleig J., Blaha P. (2023). Electronic and Ionic Effects of Sulphur and Other Acidic Adsorbates on the Surface of an SOFC Cathode Material. J. Mater. Chem. A.

[21] Hodges J.P., Short S., Jorgensen J.D., Xiong X., Dabrowski B., Mini S.M., Kimball C.W. (2000). Evolution of Oxygen-Vacancy Ordered Crystal Structures in the Perovskite Series Sr_n_Fe_n_O_3n−1_ (n = 2, 4, 8, and ∞), and the Relationship to Electronic and Magnetic Properties. J. Solid State Chem..

[22] Pérez García M. (2014). Universitat Politècnica de València. Ing. Del Agua.

[23] Ding X., Kong X., Wang X., Jiang J., Cui C. (2010). Characterization and Optimization of Ln_1.7_Sr_0.3_CuO_4_ (Ln = La, Nd)-Based Cathodes for Intermediate Temperature Solid Oxide Fuel Cells. J. Alloys Compd..

[24] Yao C., Zhang H., Dong Y., Zhang R., Meng J., Meng F. (2019). Characterization of Ta/W Co-Doped SrFeO_3−δ_ Perovskite as Cathode for Solid Oxide Fuel Cells. J. Alloys Compd..

[25] Jiang S., Sunarso J., Zhou W., Shen J., Ran R., Shao Z. (2015). Cobalt-Free SrNb_x_Fe_1−x_O_3−δ_ (x = 0.05, 0.1 and 0.2) Perovskite Cathodes for Intermediate Temperature Solid Oxide Fuel Cells. J. Power Sources.

[26] Xia W., Li Q., Sun L., Huo L., Zhao H. (2020). Electrochemical Performance of Sn-Doped Bi_0.5_Sr_0.5_FeO_3−δ_ Perovskite as Cathode Electrocatalyst for Solid Oxide Fuel Cells. J. Alloys Compd..

[27] Gao L., Zhu M., Li Q., Sun L., Zhao H., Grenier J.-C. (2017). Electrode Properties of Cu-Doped Bi_0.5_Sr_0.5_FeO_3−δ_ Cobalt-Free Perovskite as Cathode for Intermediate-Temperature Solid Oxide Fuel Cells. J. Alloys Compd..

[28] Gao J., Li Q., Sun L., Huo L., Zhao H. (2019). Enhanced Electrocatalytic Activity and CO_2_ Tolerant Bi_0.5_Sr_0.5_Fe_1−x_Ta_x_O_3−δ_ as Cobalt-Free Cathode for Intermediate-Temperature Solid Oxide Fuel Cells. Ceram. Int..

[29] Ghoneum M., Badr El-Din N.K., Alaa El-Dein M. (2024). Anti-Radiation Effect of MRN-100: A Hydro-Ferrate Fluid, in Vivo. J. Radiat. Res..

[30] Niu Y., Zhou W., Sunarso J., Ge L., Zhu Z., Shao Z. (2010). High Performance Cobalt-Free Perovskite Cathode for Intermediate Temperature Solid Oxide Fuel Cells. J. Mater. Chem..

[31] Mushtaq N., Xia C., Dong W., Abbas G., Raza R., Ali A., Rauf S., Wang B., Kim J.-S., Zhu B. (2018). Perovskite SrFe_1−x_TixO_3−δ_ (x < = 0.1) Cathode for Low Temperature Solid Oxide Fuel Cell. Ceram. Int..

[32] Zhu Z., Wei Z., Zhao Y., Chen M., Wang S. (2017). Properties Characterization of Tungsten Doped Strontium Ferrites as Cathode Materials for Intermediate Temperature Solid Oxide Fuel Cells. Electrochim. Acta.

[33] Porotnikova N., Osinkin D. (2024). Segregation and Interdiffusion Processes in Perovskites: A Review of Recent Advances. J. Mater. Chem. A.

[34] Kim D.-Y., Park C.-H., Park B.-K. (2024). Enhancing Sr-Deficient Sr (Ti_0.3_Fe_0.7_) O_3–δ_ Cathode Performance through Sm_0.5_Sr_0.5_CoO_3–δ_ Infiltration. J. Electrochem. Soc..

[35] Porotnikova N.M., Khodimchuk A.V., Zakharov D.M., Bogdanovich N.M., Osinkin D.A. (2023). Enhancement of Surface Exchange and Oxygen Diffusion of Sr_1.95_Fe_1.4_Ni_0.1_Mo_0.5_O_6–δ_ Oxide Determined by Two Independent Isotope Exchange Methods. Appl. Surf. Sci..

[36] Porotnikova N., Zakharov D., Khodimchuk A., Kurumchin E., Osinkin D. (2023). Determination of Kinetic Parameters and Identification of the Rate-Determining Steps in the Oxygen Exchange Process for LaNi_0.6_Fe_0.4_O_3−δ_. IJMS.

[37] Gao L., Li Q., Sun L., Zhang X., Huo L., Zhao H., Grenier J.-C. (2017). A Novel Family of Nb-Doped Bi_0.5_Sr_0.5_FeO_3–δ_ Perovskite as Cathode Material for Intermediate-Temperature Solid Oxide Fuel Cells. J. Power Sources.

[38] Gao L., Li Q., Sun L., Xia T., Huo L., Zhao H., Grenier J.-C. (2018). Antimony-Doped Bi_0.5_ Sr _0.5_ FeO _3−δ_ as a Novel Fe-Based Oxygen Reduction Electrocatalyst for Solid Oxide Fuel Cells below 600 °C. J. Mater. Chem. A.

[39] Liu J.-W., Li Q., Sun L.-P., Huo L.-H., Zhao H., Jean-Marc B., Sébastien F., Jean-Claude G. (2020). Tuning the High Temperature Properties of PrSrCoO_4_ Cathode with Cu^2+^ Dopant for Intermediate Temperature Solid Oxide Fuel Cells. Renew. Energy.

[40] Wang J., Lam K.Y., Saccoccio M., Gao Y., Chen D., Ciucci F. (2016). Ca and In Co-Doped BaFeO_3−δ_ as a Cobalt-Free Cathode Material for Intermediate-Temperature Solid Oxide Fuel Cells. J. Power Sources.

[41] Chen K., He S., Li N., Cheng Y., Ai N., Chen M., Rickard W.D.A., Zhang T., Jiang S.P. (2018). Nb and Pd Co-Doped La_0.57_Sr_0.38_Co_0.19_Fe_0.665_Nb_0.095_Pd_0.05_O_3−δ_ as a Stable, High Performance Electrode for Barrier-Layer-Free Y2O3-ZrO2 Electrolyte of Solid Oxide Fuel Cells. J. Power Sources.

[42] Yao C., Yang J., Zhang H., Meng J., Meng F. (2020). Cobalt-free Perovskite SrTa_0.1_Mo_0.1_Fe_0.8_O_3- δ_ as Cathode for Intermediate-temperature Solid Oxide Fuel Cells. Int. J. Energy Res..

[43] Kresse G., Joubert D. (1999). From Ultrasoft Pseudopotentials to the Projector Augmented-Wave Method. Phys. Rev. B.

[44] Kresse G., Furthmüller J. (1996). Efficient Iterative Schemes for *Ab Initio* Total-Energy Calculations Using a Plane-Wave Basis Set. Phys. Rev. B.

[45] Kulik H.J., Cococcioni M., Scherlis D.A., Marzari N. (2006). Density Functional Theory in Transition-Metal Chemistry: A Self-Consistent Hubbard U Approach. Phys. Rev. Lett..

[46] Solovyev I.V., Dederichs P.H., Anisimov V.I. (1994). Corrected Atomic Limit in the Local-Density Approximation and the Electronic Structure of d Impurities in Rb. Phys. Rev. B.

[47] Suen Y.W. (1994). Anomalous Temperature Dependence of the Correlated V = 1 Quantum Hall Effect in Bilayer Electron Systems. Phys. Rev. B.

